# Insight of Polyphenol Oxidase Enzyme Inhibition and Total Polyphenol Recovery from Cocoa Beans

**DOI:** 10.3390/antiox9060458

**Published:** 2020-05-27

**Authors:** Said Toro-Uribe, Jhair Godoy-Chivatá, Arley René Villamizar-Jaimes, María de Jesús Perea-Flores, Luis J. López-Giraldo

**Affiliations:** 1 School of Chemical Engineering, Food Science & Technology Research Center (CICTA), Universidad Industrial de Santander, Carrera 27, Calle 9, 68002 Bucaramanga, Colombia; saidtorouribe@gmail.com (S.T.-U.); jhaigo@hotmail.com (J.G.-C.); 2 Food Science & Technology Research Center (CICTA), Universidad Industrial de Santander, Carrera 27, Calle 9, 68002 Bucaramanga, Colombia; arleyvil@uis.edu.co; 3 Centro de Nanociencias y Micro y Nanotecnologías, Instituto Politécnico Nacional, Luis Enrique Erro s/n, Unidad, Profesional Adolfo López Mateos, Col. Zacatenco, C.P. 07738 Ciudad de México, Mexico; mpereaf@ipn.mx

**Keywords:** polyphenol oxidase, cocoa polyphenols, heat treatment, enzyme inactivation

## Abstract

A full factorial design (ascorbic acid/l-cysteine inhibitors, temperature, and time as factors) study was conducted to enhance inhibition of polyphenol oxidase (PPO) activity without decreasing cocoa polyphenol concentrations. The data obtained were modelled through a new equation, represented by Γ, which correlates both high polyphenol content with reduced specific PPO activity. At optimized values (70 mM inhibitory solution at 96 °C for 6.4 min, Γ = 11.6), 93.3% PPO inhibition and total polyphenol of 94.9 mg GAE/g were obtained. In addition, microscopy images confirmed the cell morphological changes measured as the fractal dimension and explained the possible cell lysis and denaturation as a result of heat treatment and chemical inhibitors. Results also showed that PPO enzyme was most suitable (higher *v*_max_/*K_m_* ratio) for catechol, with a reduction in its affinity of 13.7-fold after the inhibition heat treatment. Overall, this work proposed a suitable and food-safe procedure for obtaining enriched polyphenol extract with low enzyme activity.

## 1. Introduction

Precursors of chocolate flavor are usually obtained through enzymatic and non-enzymatic reactions, in which polyphenol oxidase, invertase, and protease are the most important enzymes [1]. Polyphenol oxidase (PPO) is a major copper enzyme [2], also known as catechol oxidase, tyrosinase, and so forth [3], and is the most important deteriorative enzyme that accelerates oxidation and degradation of polyphenols and their derivatives [4]. PPO is located in the chloroplasts [5,6] and its activation takes places during cell-damaging treatment (e.g., slicing, cutting or pulping) where oxygen is available and the local pH is not too acidic [7,8], thus causing the formation of brown pigments [2,9]. In fact, the oxygen catalyzes the enzyme reaction where the monophenols forming *o*-diphenols (monophenolase activity) and then the oxidized substrate react, producing *o*-quinones (diphenolase activity) [10,11].

The rate of enzymatic browning on food is governed by PPO action, which depends on concentration, pH, temperature, amount of phenolic compounds, and oxygen availability [3,12], thus having a different level of influence on the development of flavor, color and softening, which in turn is reflected in the loss of nutritional and quality value [13]. For instance, Misnawi et al. [7] reported that PPO of dried unfermented beans increases the polyphenol oxidation rate of (−)-epicatechin, total polyphenols, and total anthocyanidins. PPO is also susceptible during the fermentation stage; therefore, total and specific activities remaining in unfermented beans are reduced up to 1% and 9% of the original, respectively [7]. Despite the strong inactivation of PPO during fermentation, it can be regenerated during the drying process via –pH increase and uptake of O_2_^−^, and the remaining PPO activity is sufficient to catalyze oxidation of phenolic compounds [12,14]. Thereby, phenolic compound content in cocoa is affected by several factors: not only by the genetic origin, geographical and environmental conditions, but also by enzyme attack and processing conditions for chocolate production. 

Polyphenolic compounds have been widely studied, since they possess an array of nutraceutical properties for human health related to cardiovascular effects [15], antioxidant activity [16], anti-inflammatory response [17], antibactericidal effect and biological applications [18,19]. As a result of all these functional bioactivities, enriched polyphenol extracts have gained greater attention. For instance, new products such as exGrape^®^SEED, Vitaflavan from grape seeds, enriched capsules with high amounts of cocoa procyanidins (PCs), enriched dark chocolate bars (e.g., CocoaVia from Mars, and FlavaBars^®^ by Flavanaturals), and Fulyzaq™ for antiretroviral-induced diarrhea (PCs consisting of DP 3–30 from *Croton lechleri*; FDA approval) are used as a food supplement. In this sense, research focusing on inactivation of enzymes without affecting the total polyphenol content deserves further investigation.

Secondary metabolites of cocoa are purine alkaloids (e.g., caffeine, theobromine, and theophylline) and flavan-3-ols, which comprise between 0.05–1.7 wt %, and 12–18 wt % [20,21], respectively. The (−)-epicatechin constitutes the major monomeric flavanol, which also forms oligomeric and polymeric procyanidins of (epi)catechin units up to tridecamers [22]. The flavan-3-ols are also characterized as including OH groups in *ortho* position, which make an excellent substrate for PPO.

Regarding inactivation of PPO and its relationship with polyphenols, it has been studied in apricots, apples, grapes, tea leaves, potatoes, lettuce, coffee, black raisins, anthocyanidins from strawberries, catechins, quercetin, shrimps, and others [10,11]. Several inactivation strategies, for instance, using reacting enzymes [23], reducing agents (e.g., removal of oxygen using chemical agents) [2], changes on pH [24], and increasing temperature [25] have been tested. Regarding reducing agents, sulfites have been widely employed, but, currently, their use has been restricted because of their adverse effects on human health [26]. Other anti-browning agents can be used, but only a limited number are considered acceptable in terms of safety and cost to control the enzymatic browning in foods or food products [27].

l-cysteine and ascorbic acid are the most widely used inhibitor agents [24]. In fact, Pizzocaro found that ascorbic acid at 0.01–56.8 mM acted as an antioxidant reducing *o*-quinone back to the original phenol compound, while L-cysteine at 40–100 mM [2] made it possible to form complexes with *o*-quinones, and at 0.20–2.0 mM [28] it exhibited an inhibition of PPO higher than 98%. In addition, commission regulation EU No. 1129/2011 approved the use of ascorbic acid (food additive E-300) and l-cysteine (food additive E-920) in foods.

Regarding the inhibition of PPO in cocoa beans, heat inactivation at temperatures ranging from 60–98 °C, for a period ranging from 3–45 min, have been previously assayed [25,29]. For instance, Pons-Andreu et al. [29] proposed an enzymatic treatment for cocoa nibs using a blanching process. However, heating is not a valid treatment to enhance long-term inhibition of PPO activity, since the enzyme is highly thermostable [30]. In addition, several works evaluate the change of enzyme activity by qualitative color measurement (melanosis index scale) instead of measuring the specific enzymatic activity [29,31]. Furthermore, many questions remain unsolved concerning the inactivation process. None of the abovementioned studies investigated the optimal temperature, time of heat treatment, type or concentration of chemical inhibitors to enhance lower enzyme activity in cocoa beans. To our knowledge, the relationship between the total polyphenol content during the PPO denaturation and how this affects antioxidant capacity and the bioactive properties of cocoa beans has not been studied yet. 

Therefore, the aims of this work were to: (a) determine the conditions to inhibit the PPO in cocoa beans while maintaining a high level of polyphenols (to do so, concentration of inhibitor (ascorbic acid and l-cysteine), temperature, and time were evaluated); (b) develop an equation showing the relationship between PPO inactivation and polyphenol content; and (c) investigate the enzyme’s kinetic parameters and their affinity to PPO using catechol, (+)-catechin, and (−)-epicatechin as substrates.

## 2. Materials and Methods

### 2.1. Reagents

All the chemicals used were analytical reagent grade and were used without further purification. Folin–Ciocalteu reagent, gallic acid, sodium carbonate, catechol, bovine serum albumin, ascorbic acid, l-cysteine, sodium phosphate dibasic, citric acid, poly(vinylpyrrolidone) (PVP), and Coomassie brilliant blue G-250 dye were obtained from Sigma Aldrich (St. Louis, MO, USA). (+)-Catechin (≥99%), (−)-epicatechin (≥99%), and procyanidin B2 were purchased from ChromaDex Inc. (Irvine, CA, USA). Acetonitrile (HPLC-grade), ethanol (analytical-grade), and formic acid were acquired from Merck (Merck, Germany). Deionized water (18 MΩcm^−1^) from an Aqua Solution system (Aqua Solution, Inc., Jasper, GA, USA) was used for the preparation of all solutions. 

### 2.2. Recovery of Cocoa Polyphenol Extract

Fresh cocoa pods (Trinitary, clone ICS 39) were collected at Villa Santa Monica (San Vicente de Chucurí, Santander, Colombia) and immediately protected from light and transported on ice to CICTA Lab for processing. Cocoa pods are mainly composed of cocoa husk, cocoa beans, and mucilage. Thus, the cocoa beans were removed manually using a knife and the beans surrounded by mucilage were immediately removed using a mucilage remover (Penagos Ltda, Bucaramanga, Colombia). After that, the beans were immediately inactivated (as described in Section 2.3) and used for further analysis. 

### 2.3. Enzyme Inhibition

The inhibition of PPO enzyme in cocoa beans was enhanced by heat treatment. The samples were dipped in an aqueous inhibitory solution consisting of ascorbic acid and l-cysteine (1:1 *v*/*v* ratio) at same equimolar concentration. The assays were carried out as follows: 10 beans (ca. 25 g wet weight at ca. 4 °C ) were immersed into 200 mL of inhibitory solution at different concentrations (0–50 mM), times (1–5 min) and temperatures (70–90 °C) in accordance with the combinations of surface design 2^3^, which includes four replicates, a central point, and start points (Table 1). These levels of factors were chosen with the goal to maintain a high level of polyphenols; therefore, it is preferred to use a high temperature for a shorter time. Immediately after the inmersion of beans into the inhbitory solution, the samples were cooled in ice water for 30 min, and then rinsed (×3) again with deionized water (4 °C) to remove traces of ascorbic acid, and l-cysteine. Non-treated sample (fresh unfermented cocoa bean) was kept as control.

### 2.4. PPO Enzyme Extraction

The treated and non-treated beans were chopped into small pieces and homogenized. The enzyme extraction was done according to Babu et al. [32] with few modifications. Briefly, the chopped pieces were homogenized in cold extraction buffer (ratio 1:1.5 w/v, 0.01 M McIIvaine citric phosphate, pH 6.5, containing 1% PVP) during 2 min at max speed (Vortex Reax Control, Heidolph, Germany) and filtered by Whatman No. 1 filter paper (Whatman, Inc., Florham Park, NJ, USA). Homogenates were centrifuged (Heraeus, Megafuge 16R, Thermo Scientific, Waltham, MA, USA) at 10,000× *g* and 4 °C for 20 min. The resulting supernatant, called crude enzyme extract, was filtered again and used for further experiments.

### 2.5. PPO Enzyme Activity Measurement

The enzyme activity (U_PPO_) was determined spectrophotometrically according to Pizzocaro et al. [24]. The reaction mixture, containing 1.0 mL of catechol solution (0.175 M) and 2.0 mL of McIlvaine buffer pH 6.5, was added to 0.5 mL crude enzyme extract. The increase in absorbance at 420 nm (Genesys 20; Thermo Scientific-Fisher, Waltham, MA, USA) was recorded at intervals of 15 s up to 5 min at room temperature. The PPO activity was calculated by the slope of the linear portion of the curve absorbance vs. time. The enzyme activity corresponding to one unit of PPO activity was defined as the 0.001-unit change in absorbance per minute at 420 nm per mL of enzyme assay solution mixture.

The protein content of specific activity was measured according to the method described by Bradford [33]. Bovine serum albumin (BSA) was used as a standard (0–0.125 mg/mL) (*r*^2^ = 0.999). The specific activity was expressed as one unit of enzyme activity per one unit (mg^−1^) of BSA protein (U_PPO_/mg).

The percent of total inhibition was calculated as follows (Equation (1)):

(1) $$Inhibition\;\left(\%\right)=\frac{Control-Test_{i}}{Control}*100$$

where *i* is the number of the treatment according to the design. *Control* and *test_i_* were expressed as the amount of enzyme specific activity (U_PPO_/mg).

### 2.6. Substrate Kinetic Constants of PPO

The evaluation of inhibition constant was assayed using catechol, (+)-catechin and (−)-epicatechin (main catechins in cocoa) as substrate (5–200 mM) at optimal temperature for PPO activity, that is, 35 °C, as previously reported in the literature [3,28].

The reaction was modeled using the Michaelis–Menten equation (Equation (2)). The *K_m_* value and maximum velocity *v_max_* were determined using a nonlinear regression by GraphPad Prism v. 6.0 (GraphPad Soft. Inc., La Jolla, CA, USA).

(2) $$v=\frac{v_{max}*\left[s\right]}{K_{m}+\left[s\right]}$$

### 2.7. Recovery of Total Phenol Content

Recovery of phenolic compounds from non-treated cocoa beans (control sample) and beans remaining after the PPO inhibition treatment were determined as follows: beans were freeze-dried (Labconco Corp., Kansas City, MO, USA) for a final humidity <4% (according by AOAC method 931.04, 1990) [34], milled and homogenized (Grindomix GM 200, Retsch GmbH & Co., Haan, Germany). The extraction was carried out as follows: 1 g of sample was added to 60 mL of a mixture of 50% ethanol/water (w/w) at 50 °C, and stirred at 300 rpm for 30 min using a magnetic stirrer hotplate (IKA C-MAG HS7, Germany) and thermocouple (IKA ETS-D5, Germany). The resulting extract was centrifuged (5000× *g*, 4 °C, 20 min); then, the supernatant was filtered through 0.45 µm hydrophilic PTFE filter (Millipore, Milford, MA, USA), and immediately analyzed.

### 2.8. Determination of Total Polyphenol Content

The total polyphenol content of the sample was assayed using Folin–Ciocalteu reagent according to Singleton et al. [35] with modifications as follows: the reaction was initiated by the addition of 50 µL of the sample with 1.5 mL of 10-fold diluted Folin-Ciocalteu reagent. After 5 min, 1.5 mL of 7.5% (w/v) sodium carbonate was added and vortexed for 10 s. The reaction medium was stored in the dark for 1 h at 25 °C. Absorbance was measured at 765 nm (Genesys 20; Thermo Scientific-Fisher, Waltham, MA, USA) against a blank sample. A gallic acid calibration curve was prepared with 0.05–1.0 mg/mL (*r*^2^ = 0.999). Results of total polyphenols amount were expressed as mg gallic acid equivalents by gram of dried cocoa beans (mg GAE/g). 

### 2.9. Chromatographic Analysis by HPLC-DAD

The reverse phase conditions and stationary phase were optimized to detect both catechins and procyanidins in the cocoa extract. Briefly, LC was assayed on a Shimadzu (LC-2030 LT Series-i, USA) and the separation was achieved using a C18-phenyl column (4.6 × 50 mm, 2.5µm) (Xbridge, Waters, Milford, MA, USA) protected with a security guard from Phenomenex (AJ0-8788, Phenomenex, Torrance, CA, USA). The procedure consisted of water/formic acid (99.99/0.01 v/v) (solvent A), and acetonitrile/formic acid (99.99/0.01 v/v) (solvent B). The linear gradient was as follows: 0-8 min, 2% B; 8–37 min, 10% B; 37–40 min, 0% B and re-equilibrium for 10 min. The flow rate, column temperature, and diode array were set at 0.8 mL/min, 60 °C, and 280 nm respectively. Identification of both catechins and procyanidins were carried out by Ion Trap LC/MS (model 6320, Agilent Technologies, Waldbronn, Germany) equipped with an ESI source and ion trap mass analyzer, which was controlled by the 6300 series trap control software (Bruker Daltonik GmbH, V. 6.2). The mass spectrometer was operated in negative ESI mode with the following conditions: mass spectra recorded from 90–2200 *m*/*z*, nebulizer 40 psi, dry gas 12 L/min and dry temperature 350 °C. Target compounds consisted of [M−H]^−^ ions at *m*/*z* 289, 577, 865, 1153, 1442, and 1730, which correspond to monomer, dimer, trimer, tetramer, pentamer, and hexamer procyanidins structures, respectively.

### 2.10. Scanning Electron Microscopy and Image Analysis

Scanning electron microscopy (SEM) was additionally used to evaluate the microstructure of (a) non-treated cocoa beans, and (b) cocoa beans with reduced PPO activity. Beans were cut into longitudinal and transversal sections with the objective to observe their microstructure. Sections were mounted on aluminum stubs with double-sided carbon adhesive tape and observed using the XL-30 Environmental Scanning Electron Microscope (Philips, Cambridge, MA, USA) at 25 kV accelerating voltage with a BSE (backscattered electron) detector. The images were acquired in grayscale and stored in TIFF format at 712 × 484 pixels. 

Images of the samples were captured using electronic microscopy and stored as .TIF in a gray scale with brightness values between 0 and 255 for each pixel constituting the image. A generalization of the box-counting method to evaluate the fractal dimension of the images (FDt) was used. In this work, the shifting differential box-counting method was used (SDBC) [36] to evaluate the fractal dimension of texture of SEM images using the ImageJ 1.34 software. Four different images at the same magnification (1000×) were evaluated. 

### 2.11. Statistical Analysis

All measurements were repeated at least three times. Statistical analysis was done using Statistica v. 7.1 (Stat-Soft Inc., Tulsa, OK, USA). The three-way analysis of variance (three-way ANOVA) and *p*-test were used to evaluate the influence of the factors and their interactions on the experimental design. One-way ANOVA and Tukey´s multiple range test at a 5% level of significance were also evaluated. The response surface methodology, consisting of a full factorial central composite rotatable design with four replicates at the central point was conducted according to a completely randomized model. A second-order polynomial equation was used to fit the experimental data, as follows (Equation (3)):

(3) $$Y=\beta_{0}+\overset{k}{\underset{i=1}{\sum }}\beta_{i}X_{i}+\overset{k}{\underset{i=1}{\sum }}\beta_{ii}X_{i}^{2}+\overset{k-1}{\underset{\begin{array}{c} i=1\\ i<j \end{array}}{\sum }}\overset{k}{\underset{j=2}{\sum }}\beta_{ij}X_{i}X_{j}$$

where *Y* is the predicted factor, X is the extraction parameter, *β*_0_ is the value of the fitted response to the design, and *β_i_*, *β_ii_*, and *β_ij_* are the coefficients of linear, quadratic, and cross-product terms, respectively.

In this study, performance of full factorial central composite design by R-squared coefficient was measured. In addition, experimental runs were randomized to evaluate the concordance of experimental data and predicted values; therefore, the root-mean-square error (RMSE, Equation (4)) was used, as follows:

(4) $$RMSE=\sqrt[{2}]{\frac{\sum_{i=1}^{n}{\left(y_{i}-\hat{y_{i}}\right)}^{2}}{n}}$$

where $y_{i}\;$ and $\hat{y_{i}}$ is the measured value and predicted value by the model, respectively, and *n* is the number of the set data. 

## 3. Results and Discussion

### 3.1. Preliminary Inhibition Assays

Prior to optimizing the inhibition of PPO enzyme from cocoa beans, the following parameters were evaluated: (a) the nature of the inhibitor, and (b) the size of the cocoa beans. Thus, the PPO activity as a function of different inhibitory agents was determined with a solution containing 1% (w/w) ascorbic acid, 1% (w/w) l-cysteine, and mixture of ascorbic acid/l-cysteine (1:1 ratio, 1% w/w,) using heat treatment at 90 °C for 5 min as previously reported by Menon et al. [25]. At concentration >1%, inhibitors behave as quinone reducers, similar to sulfides [26]. The results showed that highest denaturation of the enzyme was enhanced by a mixture of ascorbic acid/l-cysteine (79.3%), followed by ascorbic acid (72.8%) and l-cysteine (67.5%). In addition, two sizes of cocoa beans consisting of (S_1_) chopped cocoa bean (cross section of 1 × 0.5 cm^2^), and (S_2_) whole cocoa beans, were also evaluated. Results showed that S_1_ treatment inhibited the PPO 1.2-fold more than S_2_. Interestingly, inhibition solution color after heat treatment (control system) was translucid-yellow, which was quite similar to S_2_ treatment. However, a violet color in the waste solution of S_1_ treatment was observed and could be a consequence to the greater surface contact, thus facilitating the release of polyphenols. Indeed, through HPLC-DAD-MS/MS, it was shown that loos of catechins and procyanidins (up to hexamers) on S_1_ and S_2_ treatments were 0.5 and 1.2, and 8 and 22 wt.%, respectively. 

Hence, the maximization of the inactivation of PPO was carried out using whole cocoa beans, together with a combination of ascorbic acid/l-cysteine at same equimolar concentration. 

### 3.2. Influence of Inhibition Parameters on PPO Activity

The extent of PPO inhibition as a function of treatment time, temperature and inhibitor concentration is summarized in Table 1. In addition, the recovery of total polyphenol content for each assay was evaluated (Table 1). As can be seen in Supplementary Figure S1, a non-linear relationship could be observed between values of enzyme inactivation and concentration of polyphenols (*r*^2^ = 0.60). To better understand the relationship between the two response variables, several models, such as quadratic (*r*^2^ = 0.61), exponential (*r*^2^ = 0.56), and logarithmic (*r*^2^ = 0.59) equations, were evaluated; however, none of them was able to describe the data adequately. Furthermore, a new equation (Equation (5)), which correlates high polyphenol content with reduced PPO activity—expressed in percentage—in an inverse relationship, was established as follows:

(5) $$\Gamma_{i}=\frac{\mathrm{Total}\;{\mathrm{Polyphenol}}_{i}\;\left(\%\right)}{100-\mathrm{PPO}\;{\mathrm{Inhibition}}_{i}\;\left(\%\right)}$$

where Γ—represented by the Greek uppercase letter—means high polyphenol content with low enzyme activity, as a function of % total polyphenol recovered and % PPO inhibition; i is the number of the treatment according to the design. 

**Figure 1:**
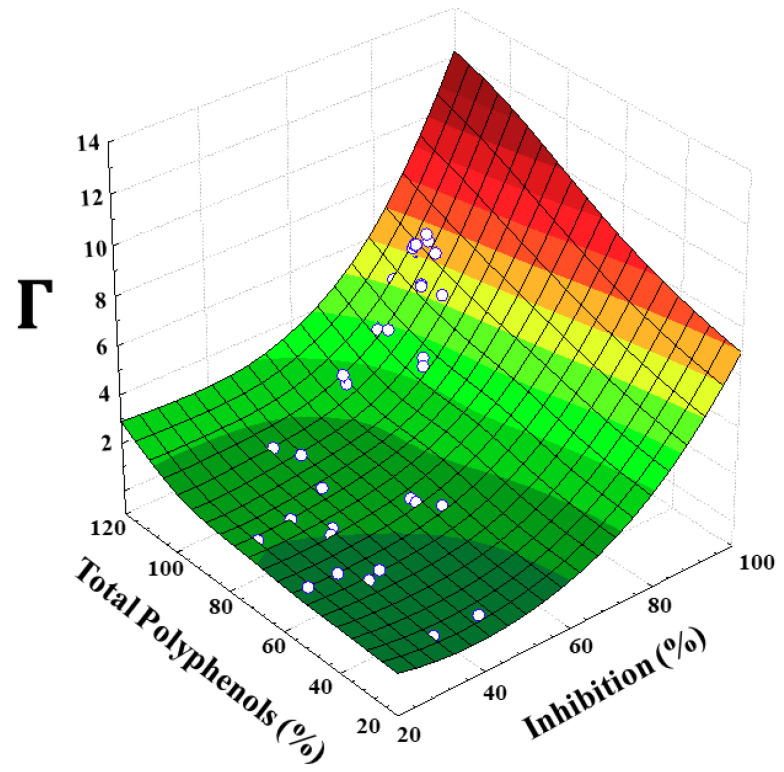
3D scheme for the correlation Γ as a function of polyphenol oxidase (PPO) inhibition (%) and total polyphenol content (%) on cocoa beans. See Equation (5).

Figure 1 shows the experimental data adjusted according to our proposed model (Equation (5)). Plotting of Γ as a function of total polyphenol (%) or inactivation (%) produced good adjustments of *r*^2^, equal to 0.91 and 0.92 respectively (Supplementary Figure S2A,B).

As can be seen, Γ had an exponential behavior; that is, with brief, low-heat treatment the rate of inactivation was lower, and both maximum enzyme inhibition and polyphenol content (lower thermal degradation of polyphenol compounds) were increased by increasing the temperature until a saturation value was reached. We hypothesized that heat treatment not only made it possible to break down the enzyme–substrate complex but also caused softening of the cell, and thereby increased the extraction yield of polyphenols. 

### 3.3. Effects of Temperature, Time and Concentration of Inhibitor on PPO Activity

Analysis of variance shows that the selected quadratic model adequately represented the data obtained for Γ with a good coefficient of multiple determination of $r^{2}$ = 0.891 (Table 2) and lower residual values (MS residuals equal to 0.748). The model’s ability to accurately predict the data based on randomly selected experiments (*n* = 15), by comparing how close predictions are to the actual outcomes, was assessed. Therefore, RMSE was 0.388, which reinforced the good performance of the model.

**Figure 2:**
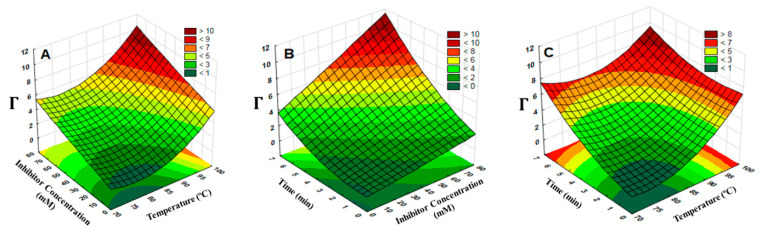
Surface response for the correlation of Γ with (**a**) temperature (T) and concentration of inhibitor (Inh), (**b**) time and Inh, (**c**) t and T. See Equation (3).

In general, ANOVA and the analysis of surface response (Table 2, Figure 2) confirmed the dependence of higher PPO denaturation as a function of the linear and quadratic effect of temperature and the linear effect of time of treatment, and concentration of inhibitor (Equation (6)). Besides, interactions between temperature with time and time with inhibitor concentration were also significant (*p* < 0.05). These can happen because (i) heat treatment affects the conformational change of the enzyme and protein–enzyme dissociation [37] and (ii) the dose-dependent inhibitory effect [2,28]. A similar trend was also observed by Oliveira and Orlanda [3], who found that the PPO enzyme was stable at temperature <67 °C, but greater denaturation of 90% could be enhanced at temperature >85 °C after 20 min [28].

(6) $$Y=54.649-1.496T+0.010\;T^{2}+1.758\;t+0.009Inh-0.023\;T*t+0.010\;t*Inh$$

At the maximum temperature (35 °C) for PPO activity, non-treated cocoa sample had a total polyphenol content of 42.1 mgGAE/g, a maximum PPO-specific activity of 32.0 ± 0.2 U_PPO_ mg^−1^, and a protein amount of 68.5 ± 3.6 mg mL^−1^, which was consistent with Lee et al. [37] and Misnawi et al. [7] with PPO-specific activity of 52 and 75 U_PPO_ mg^−1^, and total protein of 70 and 73 mgmL^−1^, respectively. Thereby, a significant effect of temperature (*p* < 0.05; Figure 2A,C) on the reduction of PPO activity, which was 1.95-, 2.23-, 2.66- and 6.35-fold lower at 70 °C, 80 °C, 90 °C, and 97 °C (compared to 35 °C), respectively, was observed. Regarding the length of treatment time (Figure 2B,C; Equation (6)), this showed the largest positive linear regression coefficient, suggesting that this factor is critical to enhancing the reduction of PPO activity. The effect of concentration of inhibitor (Figure 2A,B) was also significant (*p* < 0.05), indicating the role of ascorbic acid as an antioxidant reducing the initial *o*-quinone, and of l-cysteine as a reducing agent interfering with PPO activity before it can polymerize to melanin [3].

These findings reinforce the synergic effect of ascorbic acid and l-cysteine as an efficient solution to prevent enzymatic browning reactions. In fact, effective use of ascorbic acid and/or l-cysteine in combination with other compounds has been previously confirmed [28,38]. Based on our results, the optimum conditions to obtain the lowest enzymatic browning and highest total polyphenol content were achieved with 70 mM inhibitory solution at 96 °C for 6.4 min, for a predicted Γ = 11.8, which agreed with the experimental results obtained under these conditions, which provided a Γ = 11.6 ± 2.7, that is, 93.3 ± 2.1% PPO inhibition and total polyphenol of 94.9 ± 4.09 mg GAE/g (2.3-fold higher than non-treated samples). Under these conditions, a long-term study showed that inactivated cocoa beans (stored at 4 °C) maintain, over 2 years, their total phenol content (ca. 92 ± 3.2 mg GAE/g) and PPO activity (ca. 89 ± 3.8%) with no significant change over time (*p* < 0.05).

### 3.4. Kinetic Parameters of PPO Inhibition in Cocoa Beans

The differences in PPO activity observed varying the substrate (catechol, (+)-catechin, and (−)-epicatechin) were determined in the enzyme’s kinetic parameters. As expected, all the substrates were oxidized, displaying Michaelis–Menten kinetics [8]. The kinetic parameters calculated by nonlinear regression are summarized in Table 3. Regarding the catalytic power (*v*_max_/*K_m_* ratio), taken as an evaluation criterion, the enzyme seemed to be most suitable for small *o*-diphenols such as catechol (4440.98 U mM^−1^ mL^−1^ min^−1^), followed by (−)-epicatechin (727.38 U mM^−1^ mL^−1^ min^−1^), and (+)-catechin (637.79 U mM^−1^ mL^−1^ min^−1^). Indeed, higher affinity for catechol followed by catechin was also found by Doǧru and Erat [2].

Affinities of PPO for catechins obtained in this study were quite similar to that reported by Wuyts et al. [39] and Ho [40] with *K_m_* of 1.2 and 2.1 mM, respectively, as well as for (−)-epicatechin with *K_m_* equal to 0.65 and 1.18 mM according to Liu et al. [41] and Martinez-Cayuela et al. [42], respectively. The kinetic constant for catechol was 0.61 mM, similar to the value range from 0.18 to 0.97 mM for cocoa pulp and bean, respectively [1], but different from other plant samples, with values ranging from $7.9\times 10^{-4}$ to 203.8 mM [2,28,43], which can be due to method of extraction, nature of the subtrate, and method of measurement. 

Additionally, denatured PPO enzyme recovered using the optimized variables (70 mM inhibitory solution at 96 °C for 6.4 min) for the oxidation of catechol was also studied. Results showed that catechol had the lowest catalytic power, with 12.76 U mM^−1^ mL^−1^ min^−1^; therefore, its affinity for the substrate (*K_m_*) was reduced up to 13.7-fold. The lowest affinity of the enzyme could be a consequence of the degradation of the protein site of the enzyme and/or morphological changes in the enzyme, which can be related to its low protein content (16.04 ± 3.96 mg mL^−1^) and low specific activity (2.12 ± 0.65 U_PPO_ mg^−1^) after the heat treatment, respectively. 

Overall, these findings reinforce the high affinity of PPO for small *ο*-diphenols, and thermal enzyme denaturation, and also highlight the importance of inhibiting PPO activity for controlling the dramatic loss of polyphenols by the enzymatic action.

### 3.5. Microscopy Analysis

**Figure 3:**
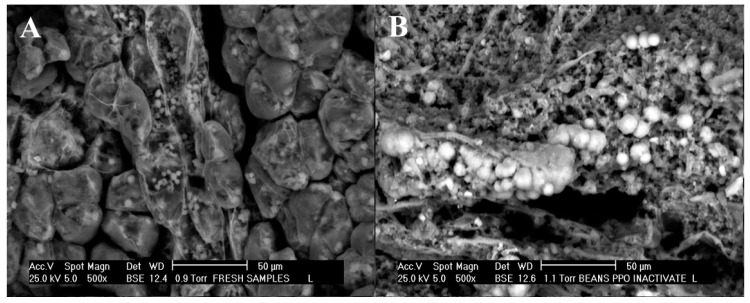
Microscopy images for the microstructure of (**A**) non-treated sample (fresh cocoa bean) and (**B**) sample after optimal conditions for the inhibition of PPO enzyme (70 mM inhibitory solution at 96 °C, 6.4 min).

An evaluation of the effect of PPO inhibition on morphology and cell wall was carried out by scanning electron microscopy. As can be seen in Figure 3A, fresh sample (non-treated sample) maintains the cell walls, which are well oval-shaped, non-fractured, solid and denser in appearance, and have the cellular content clearly embedded in them (fractal dimension of the images, FDt = 2.542). Figure 3B clearly showed evident changes after the PPO inhibition, which consisted of faster changes in temperature between 4–97 °C for short times (1–6 min), with significant differences (*p* < 0.05) observed in the FDt values. In fact, the FDt is an important tool for image analysis that makes it possible to quantify the image texture with the aim of identifying significant differences between treatments. Moreover, the cell walls become larger, smooth, fibrous and unfolded, and show evidence of more interspace and holes in the microstructure (Figure 3B) as a result of the enzyme thermal denaturation or the effect of heat shock proteins (FDt = 2.872), which are consistent with Terefe et al. [44]. This observation reinforced that heat treatment is a faster and robust method to change the conformational structure of PPO enzyme, and thus reduce its activity in cocoa beans and increase the extraction yield of polyphenols.

## 4. Conclusions

In this study, experimental conditions to inhibit the action of PPO enzyme in terms of the specific activity of PPO measurement and total polyphenol content were optimized. Our study has reported, for the first time, an equation that correlates both high recovery of total polyphenols and high inhibition of PPO enzyme in cocoa beans. Confirmation of heat denaturation during the inactivation process by SEM images, and the high affinity of PPO for small *ο*-diphenols, especially for catechol, followed by (−)-epicatechin, have also been studied. This work provides a promising, robust, easier, and food-safe procedure for obtaining enriched polyphenol extract with longer oxidative enzyme stability.

## Figures and Tables

**Table 1:** 2^3^ full factorial surface design and experimental results for the inhibition of PPO enzyme and higher polyphenol content from cocoa beans.

Run	T (°C)	Time (min)	Inhibitor [mM]	Specific Activity [U_PPO_/mg]	Total Polyphenol [mg GAE/g]	Γ *
1	90	1	50	8.97 ± 0.08	86.58 ± 2.53	3.74
2	70	5	0	18.48 ± 0.49	83.37 ± 1.45	1.68
3	63	3	25	8.40 ± 0.45	80.52 ± 3.21	3.45
4	90	1	0	20.42 ± 0.05	70.48 ± 0.90	1.05
5	90	5	0	12.30 ± 0.52	84.74 ± 4.65	2.61
6	80	3	67	6.86 ± 0.99	88.20 ± 3.43	5.07
7	80	3	25	15.05 ± 0.49	69.24 ± 1.22	1.36
8	80	3	25	18.42 ± 0.66	75.84 ± 1.34	1.38
9	80	1	25	20.08 ± 0.52	63.62 ± 4.50	0.81
10	70	1	0	21.11 ± 0.06	53.65 ± 2.32	0.41
11	70	1	50	21.15 ± 0.19	63.11 ± 1.01	0.75
12	80	6	25	5.60 ± 0.47	87.68 ± 3.21	6.14
13	70	5	50	5.17 ± 0.82	85.08 ± 2.21	6.27
14	80	3	25	14.03 ± 0.70	66.52 ±2.56	1.31
15	90	5	0	12.04 ± 0.41	85.58 ± 6.65	2.72
16	97	3	25	5.09 ± 0.36	86.32 ± 3.27	6.55
17	90	5	50	6.22 ± 0.90	85.21 ± 4.05	5.23
18	97	3	25	5.00 ± 0.31	86.73 ± 2.41	6.74
19	80	3	25	14.94 ± 0.07	68.57 ± 4.10	1.34
20	70	5	50	5.50 ± 1.14	87.67 ± 1.43	6.25
21	80	3	0	20.01 ± 0.23	71.01 ± 1.32	1.09
22	80	1	25	22.16 ± 1.14	65.75 ± 0.53	0.81
23	63	3	25	8.69 ± 0.60	79.99 ± 4.32	3.29
24	90	5	50	6.30 ± 0.41	85.15 ± 4.21	5.15
25	80	6	25	5.43 ± 0.16	87.50 ± 5.01	6.30
26	70	1	0	18.09 ± 0.04	53.24 ± 2.45	0.46
27	80	3	0	23.99 ± 0.79	74.21 ± 1.23	1.01
28	90	1	50	8.62 ± 0.86	85.60 ± 3.02	3.81
29	90	1	0	21.36 ± 0.43	74.44 ± 2.03	1.14
30	70	1	50	24.22 ± 0.26	65.94 ± 2.01	0.74
31	80	3	67	5.93 ± 0.33	82.54 ± 2.98	5.14
32	70	5	0	17.54 ± 0.78	80.84 ± 3.89	1.67

* Γ calculated according to Equation (5).

**Table 2:** ANOVA for polyphenol oxidase (PPO) inactivation through 2^3^ surface design + central points + start points. *r*^2^= 0.8083; *r*^2^adj = 0.8462.

Factor	SS	df	MS	*p*
T	8.3298	1	8.32985	0.003001
T^2^	19.4339	1	19.43392	0.000042
t	49.5296	1	49.52962	0.000000
t^2^	2.2011	1	2.20111	0.100515
Inh	35.4793	1	35.47934	0.000001
Inh^2^	0.0185	1	0.01846	0.876667
T × t	3.5258	1	3.52580	0.041099
T × Inh	0.0242	1	0.02417	0.859066
t × Inh	4.2416	1	4.24158	0.026414
Error	16.4760	22	0.74891	
Total SS	150.9190	31		

SS is the sum of the squares, df is the degree of freedom, MS is the mean square, *p* is the probability value, T is temperature, t is time, and Inh is Inhibitor.

**Table 3:** Michaelis-Menten kinetic parameters on different substrates as action of cocoa bean PPO.

Substrate	*K_m_* [mM]	*v*_max_ (U_PPO_ mL^−1^ min^−1^)	*v*_max_/*K_m_* (U_PPO_/mM mL min)
Catechol	0.61 ± 0.12 ^a^	2709 ± 21.89 ^a^	4440.98
Catechol *	8.36 ± 1.33 ^b^	106.7 ± 3.70 ^b^	12.76
(−)-Epicatechin	1.26 ± 0.37 ^a^	916.5 ± 20.59 ^c^	727.38
(+)-Catechin	1.45 ± 0.35 ^a^	924.8 ± 18.98 ^c^	637.79

* Substrate evaluated using PPO enzyme from inactivated cocoa beans at optimum conditions (70 mM inhibitory solution at 96 °C for 6.4 min). Means within a column sharing the same letter are not significantly different by Tukey (*p* > 0.05).

## Supplementary Materials

The following are available online at https://www.mdpi.com/2076-3921/9/6/458/s1, Figure S1: Relationship between specific activity of PPO and total polyphenol content on cocoa beans. Figure S2: Correlation Γ (See Equation (5) as a function a) PPO inhibition (%), and (b) Total polyphenol (%) on cocoa beans.
