# Supplementary material for: Insight of Polyphenol Oxidase Enzyme Inhibition and Total Polyphenol Recovery from Cocoa Beans
Source: Antioxidants (Basel). 2020 May 27;9(6):458. doi: 10.3390/antiox9060458 (PMC7346217; doi:10.3390/antiox9060458)

**Electronic Supplementary Material to:**

**Insight of polyphenol oxidase enzyme inhibition and total polyphenol recovery from cocoa beans**

**Said Toro-Uribe^a^, Jhair Godoy-Chivatá^b^, Arley Villamizar Jaimes^c^, María de Jesús Perea-Flores^d^, Luis J. López-Giraldo^e^***

**Figure S1:** Relationship between specific activity of PPO and total polyphenol content on cocoa beans.


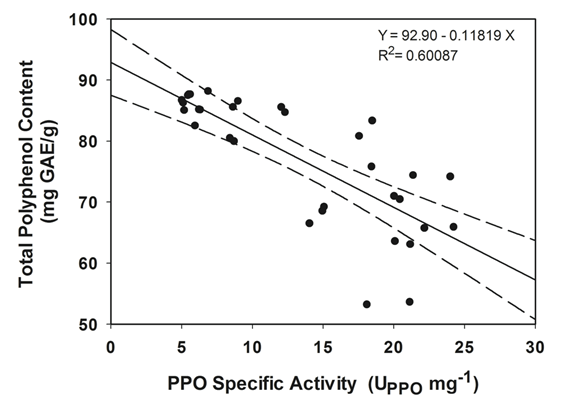


**Figure S2:** Correlation Γ (See Eq. 5) as a function a) PPO inhibition (%), and b) Total polyphenol (%) on cocoa beans**.**


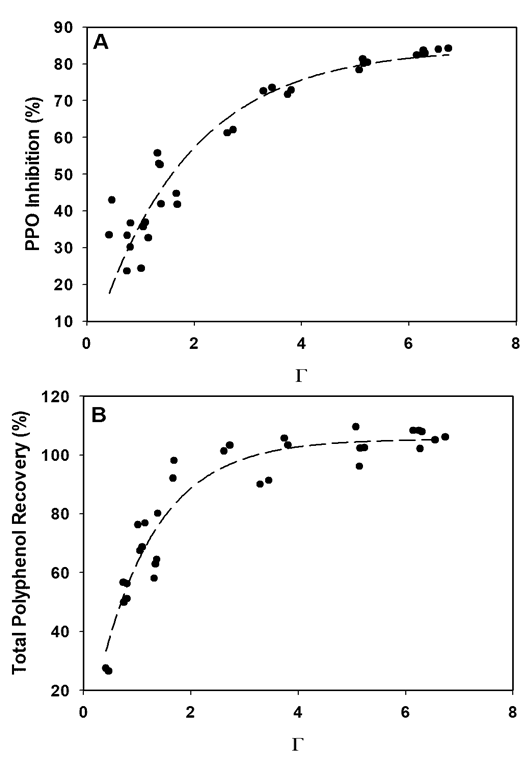

Supplement: Supplementary file 1 [file antioxidants-09-00458-s001.zip › Figure Supplement.docx]
